# Validity of two simple measures for estimating life-course socio-economic position in cross-sectional postal survey data in an older population: results from the North Staffordshire Osteoarthritis Project (NorStOP)

**DOI:** 10.1186/1471-2288-12-88

**Published:** 2012-06-27

**Authors:** Rosie J Lacey, John Belcher, Peter R Croft

**Affiliations:** 1 Arthritis Research UK Primary Care Centre, Keele University, Keele, Staffordshire, ST5 5BG, UK

## Abstract

**Background:**

Since few cohorts encompass the whole life-course, many studies that measure socio-economic position (SEP) across the life-course rely on participant recall of SEP measures from cross-sectional postal or interview surveys. It is also particularly important that SEP measures should be appropriate for the age of the population studied, as the level of missing data has been shown to increase in older people. The aim of this study was to investigate the accuracy of recall of two SEP measures in older adults, age left school and longest job, by examining their validity in a general population postal survey in North Staffordshire, UK.

**Methods:**

Sets of questions on education and longest job were included in a questionnaire at different stages of the study. All patients aged 50+ registered with three general practices were sent a baseline Health Questionnaire. 6 years later, 3410 responders were mailed a follow-up Health Questionnaire; a sub-sample of these participants took part in independent qualitative interviews. Validity was assessed by: percentage completion; internal percentage agreement within each set of questions; percentage agreement of qualitative and quantitative data for age left school and longest job; comparing recall of age left school with historical change in legal school leaving age; comparing frequency of pottery job titles with those in 1981 Census data for Stoke-on-Trent.

**Results:**

The adjusted response to different stages of the study was 71–85%. Completion of questions was 83–98%. Internal agreement was 84–97% (education) and 95–100% (longest job). Comparison of survey and interview data showed 86% agreement (± 1 year) for age left school and 91% agreement for longest job. The change in age left school data concurred with the historical shift in legal school leaving age. 11% of job titles were pottery in NorStOP data and 15% in Stoke-on-Trent Census data.

**Conclusions:**

The results from this study provide evidence for the accuracy of recall of two simple measures of SEP (age left school and longest job) in a postal survey of older adults. Consistency with evidence from external datasets indicated the potential validity of these measures for studying life-course SEP in population surveys.

## Background

From health researchers to government policy makers, the social gradient in health is a recognised challenge [1], with numerous studies providing evidence of associations between disadvantaged socio-economic position (SEP; as commonly measured by education, occupation, housing or income [2]) and higher risk of adult mortality [3-5] and unfavourable disease outcomes, for example, hip disease [6], diabetes complications [7], occupational disability from back pain [8] and atherosclerosis [9]. As the older population increases in numbers, the burden of such chronic diseases in this age group will consequently increase too and, whilst studies of SEP and health can inform policies for more successful ageing in those who have already reached older age, the influence of life-course SEP on health in older people has a wider remit in terms of the potential for intervention early in life to reduce the impact of cumulative socioeconomic disadvantage on future health [1]. Postal survey studies of life-course SEP and later health in older populations allow data collection from large populations, relatively cheaply and with minimal burden to participants, but face the following difficulties.

Firstly, in the ideal world, life-course studies measure SEP in real time, such as prospective or birth cohort studies, because they are less reliant on an individual’s recall. However, such studies are, by their nature, expensive and take many years to complete; hence there are relatively few cohorts that encompass measures of SEP across the whole life-course, such as the 1946, 1958 and 1970 UK birth cohort studies [10-12]. Instead, many studies of adults have to rely on participants’ accurate recall of SEP earlier in life using interview or postal surveys, e.g. the British Regional Heart Study, the English Longitudinal Study of Ageing, the Whitehall II Study, the British Women’s Heart and Health Study, the Retirement and Retirement Plans Survey and the West of Scotland Twenty-07 Study [13-18]. Theoretically, an interview survey should provide better recalled SEP data compared to a postal questionnaire survey because the interviewer has the opportunity ask detailed questions and clarify any misunderstandings in the interpretation of questions [19]. Indeed, a previous study that compared the accuracy of recall of social circumstances after 50 years between detailed life grid interview data and archive data concluded that “the extent to which these results depend upon life grid interviewing methods, and whether comparable results could be obtained by questionnaire, remains to be examined” [20]; in the authors’ experience, use of a questionnaire would result in the “loss of accuracy, detail and duration of lapsed time” [20]. A reduction in the accuracy of childhood SEP measures recalled in adulthood would tend to under-estimate the true associations between SEP and health outcomes [5,21].

Secondly, measurement of SEP in older people is recognised as problematic [17] since the meaning of, for example, education and occupation differs according to birth cohort [2]. In postal surveys of adults of all ages, questions about qualifications in older people can pose problems due to the potential misclassification of historical levels of educational attainment to the current equivalents, and the need to code a wide range of qualifications [17]. Similarly, a question on current or most recent job asked of a population aged 18 and over may not represent the type of work people currently aged over 50 have done for most of their working life, as they may have retired and started another job in a different area of work, and hence changed their SEP. Indeed, previous studies of disability have found no evidence of an association between current or most recent job-based adult social class and the onset of disabling pain in adults aged 50 and over [22], but a positive association between the longest job being a manual occupation and difficulty with mobility in those aged 51–61 [23], suggesting that different measures of occupation may give differing results but also that the longest job may give a better indication of cumulative lifetime occupation in older adults. In order to reduce these problems, detailed information on retrospective occupational [17] and educational histories would be preferable in the older population but the collection of this amount of data requires lengthy questionnaires which are not always feasible or acceptable in postal surveys. An additional issue is that item non-response is reported to increase with age, as shown in a US study of health risk in a cohort aged 65 and over [24] and the Finbalt Health Monitor postal survey of 20–64 year olds [25].

Thirdly, in searching for established measures of SEP used in surveys of adults in the literature, we found that some studies that included measures of education and/or occupation were conducted by interview, not by post (the English Longitudinal Study of Ageing, the West of Scotland Twenty-07 Study, the Health and Retirement Study and the Retirement and Retirement Plans Survey) [14,17,18,23]. Furthermore, studies using postal or self-administered questionnaires that included these measures did not include persons aged 80 and over in their surveys. For example, females aged 60–79 were asked questions regarding age finished full time education and longest job in a self-administered questionnaire in the British Women’s Heart and Health Study [16]; adults aged about 44 to 52 were asked the age they left secondary education and their most recent occupation in a postal questionnaire in the Aberdeen Children of the 1950s Study [21,26]; at age 52–73, males were asked the job done for the longest period of time, and at age 56–77 when their full time education ended in postal questionnaires in the British Regional Heart Study [27]; in the Whitehall II Study, civil servants aged 35–55 were asked at what age they finished full-time education [28]; and persons aged 75 and over were not required to complete the occupation or qualifications questions in the 2001 Census [29].

For our postal survey, we required short, simple measures appropriate for people aged 50 and over that would capture valid information on SEP, since there would be no opportunity for clarification with an interviewer. For the reasons outlined above, together with the view that “there is no single best indicator of SEP suitable for all study aims and applicable at all time points and in all settings” [5], we hypothesised that, in a postal survey setting, age left school and job done for most of working life would be measures of SEP specifically appropriate for use in an older population. We chose age left school as a measure of young adulthood SEP because it looks at own education; although many studies have used recall of parental education or occupation in order to measure early life SEP, these measures have been shown to be subject to some missing data [20,30] and only moderate agreement of recall [21]. We hypothesised that, in older people, recall of an individual’s own age left school would be a simple, accurately recalled measure of SEP; it would avoid the problems involved in classifying historical levels of educational attainment; and the change in school leaving age implemented in 1947 [31] could be accounted for easily in this age cohort. We chose job done for most of working life as a measure of adult SEP because in a previous study in North Staffordshire, participants aged 56 years and over had worked for a mean of 25.2 years (standard deviation 12.7 years; Lacey, unpublished data) in their main job and, as such, it would give a better indication of cumulative lifetime occupationally-based SEP in a cohort of older people. For external validation, each measure could be compared with an objective measure: the 1944 Education Act [32] for age left school and common job titles in the 1981 Stoke-on-Trent Census for job done for most of working life.

As part of a study of the epidemiology of osteoarthritis in a cohort of older adults (the North Staffordshire Osteoarthritis Project (NorStOP)), we designed a Health Questionnaire, of which the majority of the components were repeated in follow-up stages of the study; the questionnaire included a four-part question on education [33], and a five-part question about the job done for most of working life. The aim of this study was to investigate the accuracy of recall of two SEP measures in older adults, age left school and job done for most of working life, by examining their validity in a general population postal survey.

## Methods

Sequential postal Health Questionnaires (which formed part of the baseline and follow-up surveys in the population-based NorStOP study), a pre-pilot study and cognitive interviews were used as a source of information for the SEP measures to be tested (education and job done for most of working life); other measures included in the Health Questionnaire were general health, pain and socio-demographics.

### Socio-economic position measures

#### Education

Information on participants’ education was obtained by asking about age left school and education after leaving school in the Health Questionnaire [33], as follows:We would like to know about the job that you have done for most of your working life. Pease tell us:

· How old were you when you left school? ___years old. We would like to know about the job that you have done for most of your working life. Pease tell us:

· Did you go from school to full-time education or university? Y / N

· If yes, what age did you finish full-time education? ___years old

· Have you gained qualifications through study as an adult? Y / N.

#### Longest job

Information on the job that participants had done for the longest time (longest job) was obtained by asking a five-part question about “the job you have done for most of your working life” in the Health Questionnaire, as follows:

· The title of the job you have done for most of your working life (e.g. assembly worker, lawyer, office manager, shop assistant, van driver)___________________________________________________________

· The type of work of the job you have done for most of your working life (e.g. banking, building, catering, farming, office, pottery, retail)________________________________________________________________

· The year you started the job you have done for most of your working life_________

· Are you still working in the job you have done for most of your working life? Y / N

· If no, the year you finished the job you have done for most of your working life____.

This question was more detailed than the single occupation question asked in the NorStOP questionnaires at previous stages of the study [33]. Occupational data were classified according to the Standard Occupational Classification (SOC2000) [34].

### NorStOP survey

NorStOP is a large, general population-based prospective cohort study of adults aged 50 years and over at baseline in North Staffordshire, UK, conducted between 2002 and 2008. The North Staffordshire Research Ethics Committee granted approval for all stages of the study.

#### Study population

The detailed methodology and response to the baseline, 3-year and 6-year follow-up NorStOP surveys have been published previously [33,35-37]. Briefly, at baseline, all patients aged 50 years and over registered with three general practices in North Staffordshire were sent a Health Questionnaire; 7878 completed questionnaires were received, giving an adjusted response of 71.3% [35]. The adjusted responses at follow-up were 84.7% at 3 years [36] and 83.9% at 6 years [37].

#### Cognitive interview study

The questions on longest job in the Health Questionnaire were pre-piloted with 8 members of the Centre’s Research User Group in the appropriate age group. Using a cognitive interview technique, participants answered the questions in written form, and discussed their responses with RJL regarding the comprehensibility, relevance, acceptability, and comprehensiveness of the questions.

#### Qualitative study

Subsequent to the 6-year follow-up survey, a sub-sample of 27 people was interviewed as part of a qualitative study that included a reconstruction of critical landmarks in their life-course [38]. Although not planned with the current analysis in mind, the interviews provided information on age left school and longest job that could be compared with data from the surveys.

Interview transcripts were hand searched for references to age left school and job done for most of working life, and this information was entered onto a separate qualitative database by RJL, before being compared to that in the quantitative database (matched by survey identification number). If the participant had responded to the interview question “what age did you leave school” with a specific age, that age was recorded in the qualitative database; if the participant had not given an age left school but had referred to the year they left school or the age they started work, these details were also recorded. If given in the interview, information regarding jobs and length of time in the jobs (number of years or comment, e.g. “I’ve always done that” or “all my working life”) was entered into the qualitative database.

### Analysis

***Internal validity*** was analysed by

(i) Completion of questions and missing data: percentage completion by respondents in the surveys was calculated;

(ii) Internal agreement within questions: levels of expected response combinations (as percentage) were calculated within each set of questions.

***External validity*** was analysed by calculating firstly the prevalence of the self-reported SEP measures, and then

(i) Estimating agreement between the quantitative (survey) and qualitative (interview) data: as percentage, for age left school and job done for most of working life data. The job done for most of working life, and length of time in that job, reported in the qualitative interviews was calculated from the data entered into the qualitative database. From the quantitative data, the start and finish years of the job done for most of working life given in the postal questionnaire were used to calculate the number of years in the job done for most of working life;

(ii) Comparing age left school in NorStOP with the 1944 Education Act, which raised the school leaving age from 14 to 15 [32]. The change was implemented on 1^st^ April 1947 [31], affecting those born from 1st April 1933 onwards and hence those in the NorStOP cohort (date of birth range at baseline was 1903 to 1952). The age at which each NorStOP respondent reported leaving school was stratified into two groups: (1) recalled leaving school at less than or equal to age 14 years, and (2) recalled leaving school at greater than or equal to age 15 years, and plotted against year of birth, with the year in which the 1944 Education Act was implemented (1947) clearly marked;

(iii) Comparing job titles in NorStOP with 1981 Census data for Stoke-on-Trent. The prevalence of participants with SOC 2000 [34] coded longest job titles for glass and ceramics workers in the NorStOP cohort (we expected this to have been one of the most common longest jobs in this age cohort in North Staffordshire) was compared with the prevalence of coded current or most recent job titles for glass and ceramics workers in a 1981 Census Commissioned Table for Stoke-on-Trent [39]; this table, commissioned from the Office of National Statistics, was a 10% sample. In the NorStOP data, the number of the following were counted: (i) SOC 2000 occupation unit group title coded as “Glass and ceramic makers, decorators and finishers” and “Glass and ceramic process operatives”, (ii) SOC 2000 occupation unit group title coded as “Assemblers and routine operatives n.e.c.” with the Indexing word and occupational qualifier “Assembler, pottery” or “Handler”, and (iii) SOC 2000 occupation unit group title coded as “Labourers in process and plant operations n.e.c.” with “pottery” in the answers to the survey questions regarding title or type of work of job done for most of working life. For the 1981 Census data, the 1981 Census glossary was used to determine the number of occupations coded with “glass and ceramics” or “pottery” in the title. 1981 was chosen because it was the year that encompassed the start and finish dates of the job done for most of working life for 83% of the NorStOP cohort members. At baseline (2002), the minimum age in the NorStOP cohort was 50, therefore the minimum age would have been 29 in 1981. Hence, in order to align with 1981 Census data, NorStOP cohort members aged 51–90 (n = 2628) were directly compared to age 30–69 in the 1981 Census (n = 91300, after exclusion of occupations that were coded as inadequately described, not stated or other in the 1981 Census Commissioned Table for Stoke-on-Trent [39]).

### Effect of age on recall

This was analysed by calculating the number of years recall for each SEP measure; this was the time between when the SEP was measured in either the survey or the interview and the participants’ report of the age they left school or the year they started their longest job.

## Results

### Cognitive interviews

8 people completed the pre-pilot cognitive interviews and reported that the job done for most of working life questions were easy to complete, understandable, relevant and comprehensive; three people suggested rewording “area of work” to “type of work”, and two recommended repeating “job you have done for most of your working life” in each question. One person suggested asking about two jobs done for most of working life; however, a previous study of adults aged 56 years and over in North Staffordshire found only 2.3% of participants reported two or more longest jobs done for equal lengths of time (Lacey, unpublished data). Together with concerns about increasing the length of the questionnaire, and for analysis purposes, the NorStOP research team decided that asking about only one job done for most of working life was more appropriate for this age group. The questions were modified accordingly for the Health Questionnaire.

### Internal validity

(i) ***Completion of questions and missing data***

Of the 7878 responders to the baseline survey, one participant who gave their age left school as 1 was excluded, therefore the number available for this analysis was 7877. Table 1 shows that the completion of the education questions was excellent. Of 7877 participants, 97.9% completed the age left school question; of these, 7581 (98.3%) answered the “Did you go from school to full-time education or university?” question, and 7577 (98.2%) completed the “Have you gained qualifications through study as an adult?” question.

**Table 1:** Completed and missing data for education and job done for most of working life questions, stratified by age and gender

	**Age left school** (*n* = 7877)		**Full-time education after school** (*n* = 7713)		**Adult qualifications** (*n* = 7713)		**Job done for most of working life** (*n* = 2831)	
	**Completed** (*n* = 7713)	**Missing** (*n* = 164)	**Completed** (*n* = 7581)	**Missing** (*n* = 132)	**Completed** (*n* = 7577)	**Missing** (*n* = 136)	**Completed** (*n* = 2670)	**Missing** (*n* = 161)
**Gender***n* (%)								
**Female**	4327 (98.0)	89 (2.0)	4232 (97.8)	95 (2.2)	4244 (98.1)	83 (1.9)	1480 (92.6)	118 (7.4)
**Male**	3386 (97.8)	75 (2.2)	3349 (98.9)	37 (1.1)	3333 (98.4)	53 (1.6)	1190 (96.5)	43 (3.5)
**Age***n* (%)								
**50–59**	2485 (98.6)	35 (1.4)	2458 (98.9)	27 (1.1)	2459 (99.0)	26 (1.0)	1124 (97.7)	27 (2.3)
**60–69**	2313 (98.3)	39 (1.7)	2277 (98.4)	36 (1.6)	2279 (98.5)	34 (1.5)	963 (94.0)	62 (6.0)
**70–79**	1980 (97.5)	50 (2.5)	1941 (98.0)	39 (2.0)	1935 (97.7)	45 (2.3)	491 (90.3)	53 (9.7)
**80+**	935 (95.9)	40 (4.1)	905 (96.8)	30 (3.2)	904 (96.7)	31 (3.3)	92 (82.9)	19 (17.1)

Overall, the completion of the job done for most of working life questions was good. 2543/2831 (89.8%) of responders to the 6-year follow-up survey completed the job title question and 2361/2831 (83.4%) people competed the area of work question; only 122 (4.3%) of respondents left both job title and area of work questions blank (data not shown). This resulted in 2670 of responders (94.3%) with coding for a SOC 2000 occupation unit group title for their longest job (Table 1); of these, 2558 (95.8%) completed the year started longest job question and 2114 (79.2%) completed the year finished this job question; 2630 (98.5%) people completed the still working in job done for most of working life question.

The overall amount of missing data for each question was low, ranging between 1.7% and 5.7%. For each question, the proportion of missing data increased with increasing age although, even in those aged 80 and over, there was only 3.2% to 4.1% missing data for the education questions (Table 1). Compared to the education questions, the levels of missing data in the job done for most of working life question were higher, most notably in those aged 80 and over, and in females (Table 1).

(ii) ***Internal agreement within questions***

There was very good internal agreement between the education questions (Table 2). Of the 6767 people who answered “No” to going from school to full-time education or university, 97.4% correctly did not complete the age finished full time education question. Of the 814 subjects who answered “Yes” to going from school to full-time education or university, 84.0% completed the age finished full-time education question.

**Table 2:** **Agreement between responses to going from school to full-time education or university question and age finished full-time education question, by age and gender (*n*** **= 7713)**

	**“No”, did not go from school to full-time education or university**						
	**Total** (*n* = 6767)	**Female**	**Male**	**50**–**59**	**60**–**69**	**70**–**79**	**80+**
**Age finished full-time education***n* (%)							
**Age given**	177	100 (2.7)	77 (2.5)	21 (1.0)	48 (2.3)	59 (3.2)	49 (5.9)
**Left blank**	6590	3625 (97.3)	2965 (97.5)	2013 (99.0)	2036 (97.7)	1757 (96.8)	784 (94.1)
	**“Yes”, did go from school to full-time education or university**						
	**Total** (*n* = 814)	**Female**	**Male**	**50**–**59**	**60**–**69**	**70**–**79**	**80+**
**Age finished full- time education***n* (%)							
**Age given**	684	422 (83.2)	262 (85.3)	361 (85.1)	162 (84.0)	109 (87.2)	52 (72.0)
**Left blank**	130	85 (16.8)	45 (14.7)	63 (14.9)	31 (16.1)	16 (12.8)	20 (28.0)

Of those who reported not going on to full time education or university, only a small number of people (2.6%) mistakenly entered an age for the age finished full-time education question (Table 2); of these, 92% gave the same age as that at which they left school, showing that they had not gone on to full-time education or university. The likelihood of these disagreements increased with age, but was still under 6% for those aged 80 and over. Overall, 16% of those who reported going on to full-time education or university did not complete the age finished full-time education question (Table 2). Of these, those aged 80 and over were almost twice as likely to do so compared with those aged 50–79 (14.8%).

Similarly, internal agreement between the occupation questions was very good. Of the 2195 people who answered “No” to still working in the job done for most of their working life, 2083 (94.9%) answered the year finished job done for most of working life question. Of the 435 people who answered “Yes” to still working in the job done for most of their working life, only 3 (0.7%) also answered the year finished job done for most of working life question. 2060 of the 2064 (99.8%) respondents who answered both year started and year finished questions about the job done for most of their working life reported finishing the job at least one year after they started the job.

### External validity

#### Prevalence of self-reported SEP measures

Table 3 shows the self-reported prevalence of the items relating to going from school to full-time education or university, and gaining qualifications through study as an adult. Of those who answered the age left school question, 10.7% reported going on to full-time education or university, and 28.9% reported gaining qualifications through study as an adult. For both items, there was a strong age-related trend with over 75% of subjects answering each question positively being aged 50–69. Of this age group, the majority had left school at age 15 or older, whereas in those aged 70 and over, roughly half had left school at 15 or more (Table 3).

**Table 3:** Self-reported prevalence of going from school to full-time education or university, and gaining qualifications as an adult, by age and gender

**Gone from school to full-time education or university?** (*n* = 7581)							
	**Answered “Yes”**	**Female**	**Male**	**50**–**59**	**60**–**69**	**70**–**79**	**80+**
**Total***n* (%)	814	507	307	424	193	125	72
**Age left school***n* (%)							
**14 & under**	115	85 (16.8)	30 (9.8)	13 (3.1)	10 (5.2)	57 (45.6)	35 (48.6)
**15+**	699	422 (83.2)	277 (90.2)	411 (96.9)	183 (94.8)	68 (54.4)	37 (51.4)
**Gained qualifications through study as an adult?** (*n* = 7577)							
	**Answered “Yes”**	**Female**	**Male**	**50**–**59**	**60**–**69**	**70**–**79**	**80+**
**Total***n* (%)	2189	1028	1161	1089	635	346	119
**Age left school***n* (%)							
**14 & under**	313	129 (12.5)	184 (15.8)	26 (2.4)	32 (5.0)	188 (54.3)	67 (56.3)
**15+**	1876	899 (87.5)	977 (84.2)	1063 (97.6)	603 (95.0)	158 (45.7)	52 (43.7)

Overall, 16.3% (435/2670) of people who completed the job done for most of working life question at age 56 reported that they were still working in the job done for most of their working life.

(i) ***Agreement between quantitative and qualitative data***

The agreement between answers to the education and occupation questions in the postal surveys in 2002 and 2008, respectively, and information relating to the same questions gained from qualitative interviews in 2008 (after the 2008 postal survey) is shown in Table 4. In those people who gave information on age left school in both the quantitative survey and the qualitative interview, there was exact agreement in two thirds of subjects and if 1 year either side of the age left school was allowed, the agreement was 85.7%. In the remaining three cases, although the age left school agreed, one interviewee had stated “at 17, I had left school” and two had given the age they started work, which would not necessarily be the same age they left school.

**Table 4:** Agreement between quantitative and qualitative responses to the age left school and job done for most of working life questions

	**Age left school**	**Job done for most of working life**	**No. years in job done for most of working life**
Respondents giving information in Health Questionnaire* and interview**n (%)	21	22	17
**Male**	10/21 (47.6)	11/22 (50.0)	9/17 (52.9)
**Female**	11/21 (52.4)	11/22 (50.0)	8/17 (47.1)
**50–59**	8/21 (38.1)	10/22 (45.5)	5/17 (29.4)
**60–69**	8/21 (38.1)	7/22 (31.8)	7/17 (41.2)
**70+**	5/21 (23.8)	5/22 (22.7)	5/17 (29.4)
**Complete agreement***n* (%)	14/21 (66.7)	20/22 (90.9)	2/17 (11.8)
**Agreement ± 1 year***n* (%)	4/21 (19.0)	n/a	3/17 (17.6)
**Agreement ± 2 years***n* (%)	n/a	n/a	2/17 (11.8)
Longest job ≥ 29 years* and interview comment that always done job/job done for most of life**n (%)	n/a	n/a	7/17 (41.2)

*Quantitative data from postal survey Health Questionnaire.

**Qualitative data from qualitative interview study (*n* = 27).

n/a – variable not applicable to the data.

In those people who gave information on the job done for most of working life in both the quantitative survey and the qualitative interview, agreement was 91% (Table 4). In the two discordant cases, there was insufficient information common to both questionnaire and interview responses. Using only the cases in which the number of years in the longest job was given numerically, complete agreement was low (11.8%; Table 4). However, if 2 years either side of the number of years in the job was included, there was agreement in a further five cases, giving 41.2% agreement. The inclusion of comments such as “the majority of my working life” and “all of my working life” regarding a job recorded in the qualitative interview, resulted in a total agreement of 82.4%. In those aged 70 and over, 4 out of 5 showed complete agreement for the age left school question, and 100% had complete agreement for the job done for most of working life question (data not shown).

(ii) ***Comparison of age left school in NorStOP with 1944 Education Act***

**Figure 1:**
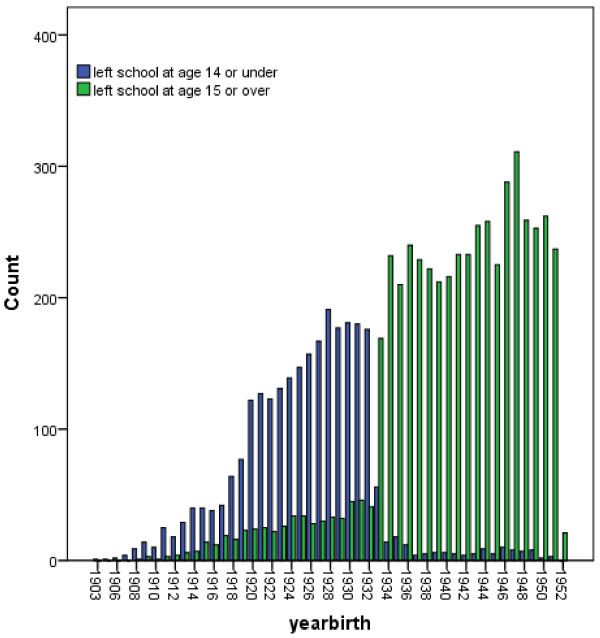
**Bar chart of self-reported age left school (≤14 vs ≥15 years) by year of birth (n = 7713).** Self-reported age left school data from the NorStOP baseline survey was stratified into two groups: left school at ≤14 years old, and left school at ≥15 years old. *The 1944 Education Act changed the legal school leaving age in England and Wales from 14 to 15, affecting those born from 1st April 1933 onwards

Figure 1 shows a bar chart of self-reported age left school stratified into two categories (left school at age 14 and under, and left school at age 15 and over) according to year of birth. The data show a clear change in the school leaving ages of people born in 1933 onwards, compared with those born before 1933, consistent with the new school leaving age in the Education Act introduced in 1947; those born before 1933 were more likely to leave school at 14 or under, whereas the majority of people born from 1933 onwards left school at 15 or over.

(iii) ***Comparison of job titles in NorStOP with 1981 Census data for Stoke-on-Trent***

Table 5 shows that, overall, 10.8% of the NorStOP cohort aged 51–90 in 2002 reported that their longest job was a glass or ceramics, or pottery, worker. In 1981, Census data showed that 14.8% of people aged 30–69 in Stoke-on-Trent reported their current or most recent job as being a glass or ceramics, or pottery, worker (Table 5). There was an age-related increase in the number of people reporting a glass, ceramics or pottery job in both the NorStOP cohort and the 1981 Census data. Similar proportions of glass, ceramics and pottery workers were found in the oldest age group in both cohorts (16.2% in NorStOP vs. 16.9% in 1981 Census), whereas smaller proportions were found in the younger age groups in the NorStOP cohort compared to the 1981 Census data.

**Table 5:** Proportion of job titles coded as glass and ceramics workers in the NorStOP cohort in 2002 and in the 1981 Census for Stoke-on-Trent

**Code**	Longest job titles coded in NorStOP cohort*, aged 51–90 in 2002	**Age**					**Code**	**Job titles coded in 1981 Census for Stoke-on-Trent, aged 30**–**69**	**Age**				
		**All**	**51**–**60**	**61**–**70**	**71**–**80**	**81**–**90**			**All**	**30**–**39**	**40**–**49**	**50**–**59**	**60**–**69**
	**Total**	2628	1185	927	448	68		**Total**	91300	26930	22430	25350	16590
**5491**	**Glass & ceramic makers, decorators & finishers**	235	83	99	46	7	**264**	**Foremen - Glass & ceramics furnacemen, kilnsetters**	50	0	30	0	20
**8112**	**Glass & ceramic process operatives**	12	4	7	0	1	**266**	**Foremen – Casters & other pottery makers**	590	130	110	210	140
**8139**	**Assemblers and routine operatives n.e.c. Assembler, pottery**	23	11	7	4	1	**271**	**Foremen – Other making & repairing glass & ceramics**	80	0	40	20	20
**8139**	**Assemblers and routine operatives n.e.c. Handler**	6	2	2	0	2	**274**	**Glass & ceramics furnacemen & workers - Glass & ceramics furnacemen, kilnsetters**	1730	440	410	470	410
**9139**	**Labourers in process and plant operations n.e.c. (with “pottery” in longest job title or area of work)**	9	5	4	0	0	**276**	**Glass & ceramics furnacemen & workers - Casters & other pottery makers**	2280	650	610	670	350
							**323**	**Other making & repairing - Glass & ceramics**	4010	900	1090	1350	670
							**423**	**Foremen - Pottery decorators**	50	0	0	30	20
							**427**	**Pottery decorators**	3050	860	930	590	670
							**541**	**Labourers & unskilled workers n.e.c. – Glass & ceramics**	1680	330	250	590	510
**Total glass & ceramics workers**		285	105	119	50	11	**Total glass & ceramics workers**		13520	3310	3470	3930	2810
**Percentage of NorStOP cohort aged 51–90 reporting longest job as a glass or ceramics worker**		10.8	8.9	12.8	11.2	16.2	**Percentage of 1981 Stoke-on-Trent Census aged 30–69 classified as a glass or ceramics worker**		14.8	12.3	15.5	15.5	16.9

*Quantitative data from postal survey Health Questionnaire. **Data from the 1981 Census Commissioned Table for Stoke-on-Trent was a 10% sample, hence the Census data presented here are grossed up by a factor of 10.

### Effect of age on recall

Table 6 is a summary of the length of time of recall of age left school and start of job done for most of working life. Age left school was recalled after between 32 and 85 years in the postal survey, and between 40 and 73 years in the qualitative interviews. The start of job done for most of working life was recalled after between 6 to 83 years in the postal survey; data for this measure was not available from qualitative interviews.

**Table 6:** Duration of recall of age left school and start of job done for most of working life

	*n*	Number of years (range)	Mean	Standard deviation
**Age left school**				
Quantitative survey	7713	32–85	51.3	10.8
Qualitative interview	18	40–73	53.1	9.1
**Start of job done for most of working life**				
Quantitative survey	2558	6–83	45.4	13.3

## Discussion

To our knowledge, this is the first study to demonstrate the accuracy of recall of SEP measures regarding age left school and longest job in a large population postal survey of older people. Good internal validity for both sets of SEP questions in the NorStOP surveys was shown by the overall low levels of missing data and the high levels of expected response combinations, indicating that the questions were acceptable and comprehensible to older people, and that the questions were measuring what they were intended to measure. Good external validity for both questions was shown by the favourable comparison of self-reported prevalences in the NorStOP surveys with the 1944 Education Act, the 1981 Census for Stoke-on-Trent and qualitative interviews in NorStOP.

Low levels of missing data reduce the likelihood of there being systematic differences between those who complete questions and those who do not in a survey [19,30]. Although the overall levels of missing data were low in the NorStOP cohort, there was a small, steady rise in missing data from age 50, except for the job done for most of working life question; from age 60, missing data for this question increased from 6% to 17% in those aged 80 and over. This compares with a study in which persons aged 75 years and over were more likely to have at least 6/174 item non-responses (including some “don’t know” and “not sure” responses) than those aged 65–74 in a questionnaire study of health risk in an elderly cohort [24]; however, when the data was restricted to the number of truly missing items, the association with age was no longer statistically significant [24]. The increase in missing data for longest job with age in the NorStOP cohort may be explained partly by gender, since females left this question unanswered more than males; the likelihood of females in this age cohort working inside the home for most of their lives might be expected to increase with age in an older population, and therefore uncertainty as to how to answer this question may have resulted in it being left blank; in this case, the wording of this question for older age groups should be amended to include time spent looking after the home to capture this information.

Although an individual’s education is often used as a generic indicator of SEP in epidemiological studies, the specific structure of the education measure, and the age at which it is asked, varies depending on the design of the study and its objective [5]. For example, in life-course studies, which often include several measures of SEP at different stages throughout life, childhood SEP frequently is measured by father’s education [9] whilst adult or current SEP is often measured by own education [7,8]. The importance of recording the level of missing data, and its implications, for these variables has been shown in a cross-sectional population survey of early life SEP in adults aged 18 and over which reported up to 20.1% missing data for parents’ highest level of education [30], and in a study of the accuracy of recall of early life SEP in 44 to 52 year olds which found only moderate agreement between father’s occupation recalled in middle-age obtained by postal questionnaire and occupational social class measured both at birth and later reported by the participant as a child [21]. This may be due to misclassification of parents’ education by their children due to variation in the meaning of terms for levels of education for different birth cohorts, although some studies have addressed this by making different categories of education specific for parental education and own education [9]. Own education, measured retrospectively as an adult, can be used as a measure of current or adult SEP but, since education specifically measures the transition from childhood SEP to adult SEP, it is also a good indicator of young adulthood SEP in life course-studies [1,2]. Our results provide evidence for the external validity of the age left school measure in adults aged 50 and over, evidenced by the clear change in school leaving age, from mainly age 14 or under to mainly age 15 or over, for those born from 1933 onwards in the NorStOP cohort, which concurred exactly with when the change in school leaving age from 14 to 15 was implemented in 1947 in England and Wales [31,32].

Occupation is frequently used to measure adult SEP by asking about either an individual’s current or most recent occupation [6,17,21,33] or their main occupation [30] or the occupation they have held for the longest time [2,13,16]. The favourable comparison of the overall proportion of glass, ceramics or pottery longest jobs in the NorStOP cohort in 2002 with the proportion of current or most recent pottery related jobs in the 1981 Census data for Stoke-on-Trent support the external validity of the job done for most of working life measure in a postal survey of older adults. One possible reason for the overall 4% higher value in the Census data is that different occupational classification systems were used, although both systems were searched in detail for coding that included terms related to pottery work. Both datasets showed a trend of decreasing number of pottery jobs reported with younger age group and, whilst the proportions of pottery related workers in the oldest age group in each cohort was almost identical, smaller proportions were found in all the younger age groups in the NorStOP cohort compared to the Census data. This is likely to be due to the decline of manufacturing industries in the UK generally, including the pottery industry in North Staffordshire, in the 1980s. Further support for the external validity of the job done for most of working life measure comes from comparison with data from a cross-sectional study of slightly younger adults in North Staffordshire, in which 25% of adults aged 50 and over stated that the job they had held for the longest time was also their current job [40]. As people approach retirement, the proportion who report still being in the job done for most of their working life would be expected to fall, due to leaving employment entirely, retiring but starting a different job, or changing job for health or other reasons. This is demonstrated in the NorStOP cohort where, at age 56 and over, 16.3% of people reported that they were still in the job done for most of their working life.

The strengths of this study are its large numbers of participants from a general population, the good responses to the postal questionnaires obtained at all stages of the study and the use of external datasets to validate the measures. The age and gender structure of the participating baseline NorStOP population was similar to that of North Staffordshire and England and Wales [35]. In addition, the NorStOP cohort included a substantial proportion of the “older old” (25.8% were aged 70 years or over and 12.4% were 80 or over at baseline), and so it was not limited to the “younger old” as in some studies [17,20]. Furthermore, our data suggests that accuracy of recall can be sustained over considerably longer periods of time than has been shown in previous studies where the accuracy of recall was examined after about 32 to 40 years [21] and after 50 years [20]; in the independent face-to-face qualitative interviews with a sub-sample of the NorStOP cohort, there was complete agreement with age left school survey data recalled after a maximum length of 73 years.

The main weakness of our study is that the measures of external validity were “ecological”. We demonstrated that recalled school leaving age was consistent with contemporaneous national changes in this age, but had no source of alternative independent validation of each individual’s recalled school leaving status. We showed that the pattern of recall of one common major occupation in the North Staffordshire area was consistent with the frequency of that occupation in the population of North Staffordshire as it appeared in Census records from a year that represented the working age of our surveyed population, but we had no source of independent validation of each individual’s recalled main job during working life. Since recall of these two items provide such potential for population surveys to provide measures of life-course SEP, future studies which could provide independent validation of these measures at the individual level would be useful. It should also be noted that, within the limitations of the study, the two SEP measures would be generalisable only to general populations aged 50 and over.

## Conclusions

The results from this study provide evidence for the accuracy of recall after up to 73 years of two simple measures of SEP, age left school and longest job, in a postal survey of older adults including the oldest old. Agreement within sets of questions and consistency with evidence from external datasets indicated the potential validity of these measures for use in studying life-course SEP in cross-sectional population surveys of older people.

## Competing interests

The authors declare that they have no competing interests.

## Authors’ contributions

RJL developed the idea for the analysis, conducted the literature review, co-ordinated the study, performed the analysis and drafted the manuscript. JB performed the analysis and assisted in the drafting and reviewing of the manuscript. PRC designed the original North Staffordshire Osteoarthritis Project and was its principal investigator, and assisted in the drafting and reviewing of the manuscript. All authors read and approved the final version of the manuscript.

## Pre-publication history

The pre-publication history for this paper can be accessed here:

http://www.biomedcentral.com/1471-2288/12/88/prepub
